# Evaluation of Shear Bond Strength of Methacrylate- and Silorane-based Composite Resin Bonded to Resin-Modified Glass-ionomer Containing Micro- and Nano-hydroxyapatite

**Published:** 2016-06

**Authors:** Farahnaz Sharafeddin, Marzie Moradian, Mehran Motamedi

**Affiliations:** 1 Dept. of Operative Dentistry and Biomaterials Research Center, School of Dentistry, Shiraz University of Medical Sciences, Shiraz, Iran.; 2 Postgraduate Student, Dept. of Operative Dentistry, School of Dentistry, Shiraz University of Medical Sciences, Shiraz, Iran.; 3 Dept. of Operative Dentistry, School of Dentistry, Shiraz University of Medical Sciences, Shiraz, Iran.

**Keywords:** Bond Strength, Composite Resin, Glass-ionomer Cement, Hydroxyapatite

## Abstract

**Statement of the Problem:**

The adhesion of resin-modified glass-ionomer (RMGI) to composite resin has a very important role in the durability of sandwich restorations. Hydroxyapatite is an excellent candidate as a filler material for improving the mechanical properties of glass ionomer cement.

**Purpose:**

The aim of this study was to assess the effect of adding micro- and nano-hydroxyapatite (HA) powder to RMGI on the shear bond strength (SBS) of nanofilled and silorane-based composite resins bonded to RMGI containing micro- and nano-HA.

**Materials and Method:**

Sixty cylindrical acrylic blocks containing a hole of 5.5×2.5 mm (diameter × height) were prepared and randomly divided into 6 groups as Group 1 with RMGI (Fuji II LC) plus Adper Single Bond/Z350 composite resin (5.5×3.5 mm diameter × height); Group 2 with RMGI containing 25 wt% of micro-HA plus Adper Single Bond/Z350 composite resin; Group3 with RMGI containing 25 wt% of nano-HA plus Adper Single Bond/Z350 composite resin; Group 4 with RMGI plus P90 System Adhesive/P90 Filtek composite resin (5.5×3.5 mm diameter × height); Group 5 with RMGI containing 25 wt% of micro-HA plus P90 System Adhesive/P90Filtek composite resin; and Group 6 with RMGI containing 25 wt% of nano-HA plus P90 System Adhesive/P90 Filtek composite resin. The specimens were stored in water (37° C, 1 week) and subjected to 1000 thermal cycles (5°C/55°C). SBS test was performed by using a universal testing machine at a crosshead speed of 1 mm/min. Data were analyzed by two-way ANOVA and Tukey test (*p*< 0.05).

**Results:**

There were significant differences between groups 1 and 4 (RMGI groups, *p*= 0.025), and groups 3 and 6 (RMGI+ nano-HA groups, *p*= 0.012). However, among Z350 and P90 specimens, no statistically significant difference was detected in the SBS values (*p*= 0.19, *p*= 0.083, respectively).

**Conclusion:**

RMGI containing HA can improve the bond strength to methacrylate-based in comparison to silorane-based composite resins. Meanwhile, RMGI without HA has the best bond strength to silorane-based composite resins.

## Introduction

Currently, the most common tooth-colored restorative materials used for esthetic purposes are composite resins. However, recurrent caries caused by polymerization shrinkage is a major disadvantage.[1] Several restorative methods have been offered to solve or at least reduce this problem, such as the use of liners beneath the restoration,[2] incremental placement of restorative material,[3] increasing the filler content of the composition, and lately, use of ring-opening monomers.[4] Efforts have been made to improve the clinical performance of methacrylate-based composite resins, which has led to the development of new monomers such as ring-opening silorane and new filler technology such as nano fillers.[5]

Siloranes, a new class of ring-opening monomers, were produced to overcome the difficulties related to polymerization shrinkage. This new type of monomer is derived from the reaction of oxirane and silorane molecules with a volumetric shrinkage determined to be 0.99 volume%. Opening and extending of the oxirane ring during polymerization in this new system would compensate the volume reduction.[6] Filtek Z350 is a nanofilled composite resin with 65‒75 wt% of silica and zirconia nanofillers,[7] which is claimed to have a low shrinkage because of its high filler content.[8]

The use of a liner with lower elastic modulus or a base with fluoride release property is a clinical approach to decrease the polymerization shrinkage.[9] Lamination of dentin with glass-ionomer cement (GIC) is strongly recommended to enhance the adhesion to dentin and minimize the microleakage, particularly when the margin of the restoration is on the dentin.[10]

Fluoride release, adhesion to mineralized dental tissues, and a coefficient of thermal expansion similar to that of tooth structure are some of the advantages of GIC that make it possible to be used as an alternative layer of dentin in the composite resin fillings as well as the main filling material.[10-11] However, sensitivity to desiccation and moisture and its poor mechanical properties of GIC[12] have prompted researchers to find solutions to overcome such disadvantages.[13] In one research, the use of amalgam, silver and metal powders as reinforcements in GIC powder was suggested; however, these products have inferior esthetic appearance and decrease bond strength to enamel.[14] Incorporation of a light-cured catalyst and resin into light-cured GIC can accelerate the setting reaction and improve the mechanical strength of this material.[15] Resin-reinforced GIC is extraordinarily high in flexural strength despite having lower compressive strength than the conventional GIC.[16] Mechanical strength could be improved by incorporating SiC whiskers or short fibers into GIC,[17] but very small fibers may reside in vital organs and jeopardize their health like what asbestos fibers do.[13]

Hydroxyapatite (HA), a calcium phosphate, has a chemical composition and a crystal structure similar to apatite in tooth structure and in human skeletal system. It also offers excellent biological behavior and is the principal mineral component of the enamel, comprising more than 60% of dentin by weight. As GICs have been found to interact with HA via the carboxylate groups in polyacid, the incorporation of HA into GICs may not only improve the biocompatibility of GICs, but also might have the potential to improve its mechanical properties. The bond strength may also increase regarding a composition similar to that of enamel and dentin.[18]

Several studies investigated the effect of adding different amounts and sizes of apatite powder to GIC for improving the physical property of this cement.[19-22] These studies demonstrated that GICs containing hydroxyapatite exhibit better mechanical properties and higher bond strength to dentin than the conventional GICs. A study reported that hydroxyapatite-reinforced glass-ionomer, 75wt% of glass-ionomer and 25wt% of hydroxyapatite, exhibited the highest bond strength to dentin.[23] It has been demonstrated that adding nano-hydroxyapatite (nano-HA) to glass-ionomer shows higher bond strength to tooth structure compared to micro-hydroxyapatite (micro-HA). The decreased size of nano-HA particles, similar to that of the minerals in tooth, leads to increased surface area and higher solubility, filling the enamel defects with higher performance ;this phenomenon occurs through releasing calcium and phosphate ions and by increasing the bond strength between the tooth and the restorative material.[24]

In all the cases where the glass-ionomer is employed as a base or liner, the adhesion of this base or liner to the restorative material (particularly composite resin) has a very important role in retention, durability, and strength of the restoration. As no study was carried out on the bond strength of resin-modified glass-ionomers (RMGI) containing micro-HA and nano-HA to composite resin, the aim of this study was to evaluate the SBS of nanofilled and silorane-based composite resins bonded to RMGI containing micro- and nano-hydroxyapatite. 

## Materials and Method

In this experimental study, 60 specimens were prepared. The tested powders were prepared by mixing micro-HA (Sigma-Aldrich Inc.; USA) and nano-HA particles (Sigma-Aldrich Inc., USA) with RMGI powder (GC; Tokyo, Japan). The two powders prepared in this study included the glass-ionomer powder reinforced with 25 wt% of micro-HA powder,[23] and the glass-ionomer powder reinforced with 25 wt% of nano-HA powder. Thus, the micro-HA and nano-HA powders were weighed carefully by using an electronic weighing machine (AND; GR+360, Japan) with a precision of 0.001. The correct ratio was added by weighing the glass powder. In order to obtain a uniform powder in the samples, after initial mixture by hand, the mixed powders were placed in amalgam capsules in an amalgamator (Ultramat 2; SDI, Australia) for 20 seconds. Resin-modified glass-ionomer powder (Fuji II LC) with no additives was used to prepare the control samples.

Sixty acrylic (Acropars; Iran) blocks were prepared by using a metal cylinder mold measuring 25×25 mm. The resin blocks were polished smooth with 220-, 320- and 400-grit abrasive paper. A hole, 5.5 mm in diameter and 2.5 mm in height, with retentive grooves was prepared at the center of the polished surface using a #556 diamond fissure bur.[25] Samples were divided into 6 groups, each with 10 samples as follows.

In Group 1, the powder and liquid of RMGI was mixed on a wide surface of glass slabs with a powder-to-liquid ratio of 3.2gr:1gr and placed in the cavity embedded in the mold. To acquire a smooth surface without bubbles, a sheet of celluloid plastic was placed on the surface and then a glass plate was placed on it. Then the samples were cured for 20 seconds with an LED light-curing unit (Demi Plus; Kerr, Switzerland) at a light intensity of 1200 mW/cm[2] according to the manufacturer’s instructions. The glass plate and celluloid tape were carefully removed and then a layer of Adper Single Bond 2 (3M ESPE; USA) was applied to the surface by using a micro-brush, gently air-dried, and then cured for 20 seconds. A Teflon cylinder, 5.5 mm in diameter and 3.5 mm in height, was used for the preparation of composite molds. Nano-composite resin (Filtek Z350; 3M ESPE, USA) was added to the RMGI surface in two layers measuring 1.5×2 mm and cured for 40 seconds. In Group 2, the glass powder in this group contained 25 wt% of micro-HA powder and the other procedures were similar to group 1.In Group 3, the glass powder in this group contained 25 wt% of nano-HA powder and the rest of procedures were similar to group 1.In Group 4, powder and liquid of RMGI was mixed similar to that in group 1. The silorane self-etching primer (3M ESPE; USA) was placed on the surface of glass with a micro-brush and exposed to a gentle air stream, followed by curing for 10 seconds. Adhesive bond (3M ESPE; USA) was placed with a micro-brush and then exposed to a gentle air stream, followed by curing for 20 seconds. A Teflon cylinder, 5.5mm in diameter and 3.5mm in height, was used for the preparation of composite resin molds. Silorane-based composite resin (P90; 3M ESPE, USA) was added to the RMGI surface in two layers of 1.5×2 mm and cured for 40 seconds. In Group 5, the glass powder in this group contained 25 wt% of micro-HA powder and the other procedures were performed similar to those in group 4.In Group 6, the glass powder in this group contained 25 wt% of nano-HA powder and the other procedures were performed similar to those in group 4.

**Figure 1:**
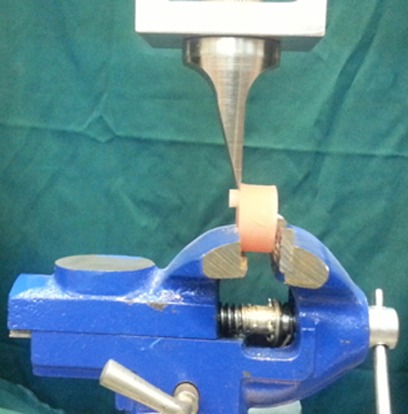
The specimen under the SBS test in the universal testing machine.

All the procedures were performed by the same operator. The samples were stored in distilled water for 1 week in an incubator (Binder; 7200 Tuttlingen, Germany) at 37°C. Each group was thermocycled 1000 times at 5±2/55±2°C for 30 seconds under every temperature, with 30 seconds for the transfer of each specimen. A shearing force was applied to the samples in a special fixture using a knife-edge blade in a universal testing machine (Zwick/Roell; Z020 Germany) at a crosshead speed of 1 mm/min until failure occurred (Figure 1). Then, the load values headed to failure were recorded.

Statistical analyses were performed with SPSS software (version 15) using two-way ANOVA and Tukey test. The level of significance was set at *p*< 0.05.

## Results

**Figure 2:**
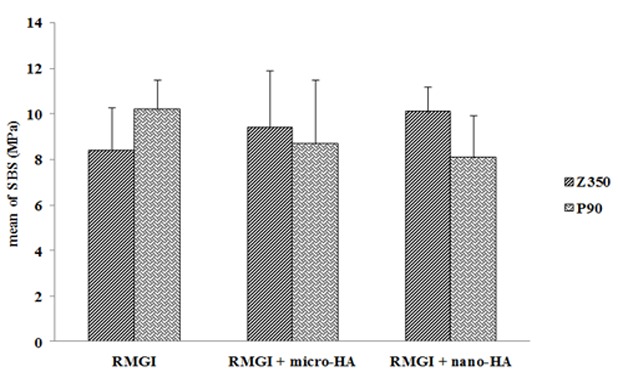
Comparison of shear bond strength of Z350 and P90 bonded to three types of RMGI

The SBS values are given in Table 1. Among Z350 specimens, SBS value for RMGI plus nano-HA (group 3) was higher than the other groups, with no significant difference (*p*=0.19). Among P90 specimens, RMGI (group 4) had the highest SBS value, with no significant difference, though (*p*= 0.083). The results of Tukey test showed significant differences between groups 1 and 4 (control, *p*= 0.025), groups 3 and 6 (RMGI plus nano-HA, *p*= 0.012). Yet, no significant difference was noted between groups 2 and 5 (RMGI plus micro-HA, *p*= 0.538). (Figure 2)

**Table 1:** The means and standard deviations of shear bond strength values (MPa) of Z350 and P90 to three types of RMGI.

**Glass-ionomer cement**	**Composite resin**	
	**Z350 (mean±SD)**	**P90** **(mean±SD)**
RMGI	8.4±1.9^A,a^	10.2±1.3^A,b^
RMGI + micro-HA	9.4±2.5^A,a^	8.7±2.8^A,a^
RMGI + nano-HA	10±1.12^A,a^	8.06±1.9^A,b^

Differences in capital letters indicate statistically significant differences within columns, and differences in lowercase letters indicate statistically significant differences within rows (*p*< 0.05). RMGI: Resin-modified glass-ionomer, HA: Hydroxyapatite.

## Discussion

Hydroxyapatite has attracted considerable attention as a biomaterial for dental applications due to its similarity in crystallography and chemical structure to that of human hard tissues, i.e. tooth and bone. A number of studies[19-22] have evaluated the effect of adding HA powder to restorative dental materials such as GIC, reporting that the presence of HA in the GIC matrix improves the mechanical properties and bond strength to tooth structures.

The use of GI as a base under composite resin restoration is recommended as an effective method to decrease microleakage. In the sandwich technique, the bond between GI and composite resin is one of the main factors in retention, durability, and sealing of the restoration.[26] A large number of studies have shown that RMGI has significantly higher mechanical properties and bond strength compared with conventional GI.[27] A study revealed that RMGI generally has much higher flexural strength compared to conventional GIC.[28] Pamir *et al.* and Taher *et al.* reported that the bond strength of composite resin to the RMGI was considerably higher than that of conventional GIC.[10, 29] It has been suggested that a similarity in the compositions of these two materials and curing mechanisms by the free-radical initiator system might be responsible for the enhanced bond strengths.[25] Therefore, in the present study, the RMGI containing HA was employed.

Since etch-and-rinse systems require separate rinsing and drying steps, they have high technique sensitivity and might weaken the GI and produce micro-cracks during drying of its surface.[30] In this study, etching procedure was not accomplished with adhesive resin.

Among Z350 specimens of current study, SBS value for RMGI containing nano-HA was higher than all other groups; though no statistically significant difference was found. Among the silorane-based composite resin specimens, the highest bond strength was achieved in the control group, with no significant differences between the SBS values.

To achieve good interfacial contact, the adhesive must have a low surface tension and the substrate should have a high surface energy. Among dental materials, GIC is high-energy substrates.[31] Therefore, incorporating HA into glass-ionomer powder leads to wider particle-size distributions,[24] and consequently higher surface energy. This might be the reason why the SBS value of Z350 with RMGI containing HA is higher than that of RMGI. On the other hand, higher bond strength of RMGI containing nano-HA in the Z350 group compared to micro-HA group might be attributed to smaller particle size of nano-HA and highly crystalline structures that increase the surface free energy.

One of the factors influencing the bond strength is the viscosity of bonding agent.[26] Mount reported that higher bond strength between the composite resin and RMGI was attained with a decrease in the viscosity of the bonding agent as a result of lower contact angle, which lead to better wetting of the surface by the bonding agent.[32] Comparison of the two bonding agents in the present study showed that the lower viscosity of Adper Single Bond compared with Silorane Adhesive explained the higher bond strength of this bonding with RMGI containing HA. The lower bonding performance of silorane to RMGI containing HA might be attributed to low surface wettability of the intermediate resin adhesive and high surface free energy of RMGI containing HA.

A few studies investigated the bonding of silorane-based composite resin to RMGI. Boushell *et al.* reported no significant difference in the shear bond strength of silorane-based composite resin (Filtek LS) to RMGI versus Z250/Adper Scotch Bond SE.[33] It has been reported that the silorane composite resin had significantly lower bond strength to RMGI compared to methacrylate composite resin.[34] But in the current study, silorane composite resin exhibited higher bond strength to RMGI in comparison to Z350. The difference observed in this study might be related to the use of self-etch adhesive system with methacrylate composite resin in the above-mentioned studies; whereas, in this study no etching system was used with Z350 specimens and Adper Single Bond was applied alone. Silorane adhesive system is considered a mild self-etching adhesive due to its high pH value (2.7). It can create mechanical interlocking between the bonding agent and the porosity caused through mildly etching of the RMGI surface.[35] Consequently, silorane adhesive system can increase the bond strength in comparison with the Adper Single Bond which includes no etching procedure. In this regard, Kasraie *et al.* showed that application of the self-etch system resulted in greater increase in micro shear bond strength between the RMGI and composite resin compared with the use of etch-and-rinse system.[26]

Hydroxyapatite may serve as a form for additional chemical interaction with the adhesive functional monomer.[36] But in this study, the self-etch adhesive of silorane containing phosphate-based functional monomer[37] did not improved the bond strength with RMGI containing HA compared with the control group. Zhang *et al.*[38] showed that the chemical interaction with hydroxyapatite strongly depended on the aggressiveness of the adhesive. They indicated that the strong Adper Prompt L-pop (pH~0.8) experienced a higher degree of chemical reaction with HA than the mild Adper Easy Bond (pH~2.5). Therefore, the HA content has considerably less positive effect on the self-etch adhesive of silorane.

The buffering effect of HA on self-etching monomers might change the acidity of the adhesive and the activity of a self-etching adhesive might be inhibited by a neutralizing reaction with HA.[36] Therefore, it seems that the presence of HA in RMGI and greater solubility of nano-HA compared to micro-HA can reduce the acidity of mild self-etching silorane and decrease the mechanical retention despite the possibility of chemical bond with the functional monomer.

Pairwise comparison of the groups in the present study showed significant differences between RMGI + nano-HA groups. Presence of HA particles increases the surface free energy of RMGI containing HA and decreases the achievable surface resin layer for bonding to composite resin. On the other hand, self-etching primer of silorane can also change the free surface energy in RMGI containing HA, which leaves an undesirable effect on the bonding. These changes can contribute to lower bond strength in P90 specimens.

The technique of a glass-ionomer sample preparation can affect the bond strength results.[39-40] In this study, the surface of RMGI was cured through a glass plate and a sheet of celluloid plastic to produce a smooth surface without bubbles, which would lead to lower bond strength compared with the clinical situation. A smooth and glazed cement surface under composite resin cannot be reproduced in clinical conditions; thus, the air-inhibited layer is preserved and increases the bond strength of RMGI to composite resin in the clinical situation.

This study was performed in an attempt to improve the bond strength between the methacrylate- and silorane-based composite resin and RMGI by reinforcing the RMGI with HA particles. Due to the lack of literature on the bonding strength of RMGI containing HA to composite resin, further studies are necessary to evaluate the effectiveness of different ratios of HA in different GICs. 

## Conclusion

Under the limitations of this *in vitro* study, it can be concluded that mixing micro-HA and nano-HA particles with RMGI powder does not have a significant effect on the bonding of RMGI to nano composite and silorane-based composite resin. Moreover, it may be inferred that under clinical conditions, RMGI containing HA can improve the bond strength to methacrylate-based composite resins; while, RMGI without HA has the best bond strength to silorane-based composite resins.
